# MSclassifier: median-supplement model-based classification tool for automated knowledge discovery

**DOI:** 10.12688/f1000research.25501.1

**Published:** 2020-09-10

**Authors:** Emmanuel S. Adabor, George K. Acquaah-Mensah, Gaston K. Mazandu

**Affiliations:** 1 School of Technology, Ghana Institute of Management and Public Administration, Accra, Ghana; 2 Pharmaceutical Sciences Department, Massachusetts College of Pharmacy and Health Sciences, Worcester, MA, USA; 3 African Institute for Mathematical Sciences and Computational Biology Division, Department of Integrative Biomedical Sciences, Institute of Infectious Disease and Molecular Medicine, University of Cape Town, Cape Town, South Africa

**Keywords:** Breast cancer, protein subcellular localization, machine learning, software package, HER2 receptor status, classification.

## Abstract

High-throughput technologies have resulted in an exponential growth of publicly available and accessible datasets for biomedical research. Efficient computational models, algorithms and tools are required to exploit the datasets for knowledge discovery to aid medical decisions. Here, we introduce a new tool, MSclassifier, based on median-supplement approaches to machine learning to enable an automated and effective binary classification for optimal decision making. The MSclassifier package estimates medians of features (attributes) to deduce supplementary data, which is subsequently introduced into the training set for balancing and building superior models for classification. To test our approach, it is used to determine HER2 receptor expression status phenotypes in breast cancer and also predict protein subcellular localization (plasma membrane and nucleus). Using independent sample and cross-validation tests, the performance of MSclassifier is evaluated and compared with well established tools that could perform such tasks. In the HER2 receptor expression status phenotype identification tasks, MSclassifier achieved statistically significant higher classification rates than the best performing existing tool (90.30% versus 89.83%, p=8.62e-3). In the subcellular localization prediction tasks, MSclassifier and one other existing tool achieved equally high performances (93.42% versus 93.19%, p=0.06) although they both outperformed tools based on Naive Bayes classifiers. Overall, the application and evaluation of MSclassifier reveal its potential to be applied to varieties of binary classification problems. The MSclassifier package provides an R-portable and user-friendly application to a broad audience, enabling experienced end-users as well as non-programmers to perform an effective classification in biomedical and other fields of study.

## Introduction

Machine learning tools are required to solve binary classification problems for optimal decision making in medicine and other fields of study. In recent times, they have been used to predict subcellular localization of proteins to assist in the functional annotation of gene products and protein secondary structure ^1, 2^. As the identification of the subcellular location of any given protein provides insights into its function, this prediction task is highly valuable. This is more so as the specific functions of many proteins remain to be fully characterized. In other contexts, for instance medicine, classifications of patients in breast cancer and other diseases are important for administering therapies. There are five molecular sub-types of breast cancer identified: basal-like, Luminal A, Luminal B, human epidermal growth factor receptor 2- (HER2-) enriched, and normal-like ^3^. The prognosis and administration of therapies in breast cancer is aided by the determination of molecular subtype phenotypes ^4^.

However, for various reasons, occasionally immunohistochemistry and other methods for establishing the presence or absence of these receptors do not necessarily cover all available samples. For example, results can be equivocal for some samples. Machine learning techniques can be trained with data from those samples that have been definitively characterized to correctly classify other uncharacterized samples’ phenotypes based on gene expression profiles. Machine learning methods rely on availability of large datasets to infer accurate outcomes for appropriate decisions concerning problems. With the advent of DNA microarray and next generation sequencing technologies, huge amounts of data are increasingly becoming available for use by these machine learning methods. These have permitted machine learning methods to be applied to characterize prognostic breast cancer samples for constructing patient-specific networks and disease groupings in precision medicine ^5– 7^.

Machine learning methods based on Random Forest have been used to identify a gene regulatory program of human breast tumour progression ^8^. Other methods such as Support Vector Machine and Naive Bayes, have all been applied to studies in breast cancer ^9^. Other methods applicable to such problems are Logistic Regression, Bayesian Networks, K-nearest neighbours and tree-based methods ^10– 12^. In general, binary classification problems, such as breast cancer classification, commonly occur in nature and they rely on these machine learning methods for effective grouping, and the classification of multiple outcomes.

These methods are implemented in software packages/applications. For instance, several of these methods are implemented in the Weka package ^13^. In R, implementations are provided as fitting functions as well as packages such as randomForest ^14^, ISLR ^15^ and e1071 ^16^ among others. Unlike linear regression models, which predict quantitative response variables, these methods infer models to predict qualitative response variables.

Recently, median-supplement approaches were introduced and found to outperform the traditional machine learning methods in binary classification models involving classification of receptor status phenotypes in breast cancer ^17^. More importantly, these approaches achieve accuracies that compare favourably with other protein/mRNA-based procedures to decipher hormone and HER2-receptor status phenotypes in as much as they outperform traditional machine learning methods ^17^. This implies that irrespective of the performance of the traditional methods, enhanced approaches provide better results in binary classification problems. However, none of the existing packages (implementations) supports the new median-supplement approaches to the binary classification problems.

Here, we aim to provide a median-supplement based tool, MSclassifier, for automated knowledge discovery from data and illustrate its applicability to both breast cancer and other binary classification problems in broader contexts of study. This provides an effective binary classification tool, preventing biases that may originate from requirements of traditional tools which generally influence the classification decisions. It enhances the capacities of both Naive Bayes and Random Forests to infer models that provide more accurate predictions of classes of observations. This package is implemented in R under free software (GNU General Public Licence).

In performing an effective binary classification, MSclassifier introduces a predetermined number of supplementary instances based on the median of each attribute (feature) of the training sets for binary classification problems involving unequal members of classes. The supplementary instances along with the training instances form a new set from which a Naive Bayes or a Random Forest model is inferred to predict new instances. The provision of additional instances introduced by the new methods increases their prediction accuracies. This is because the effectiveness of the learning methods is improved whenever the training instances are more ^18^. This has necessitated the design of the software package presented in this report. There are existing tools in R, namely randomForest ^14^ and e1071 ^16^, which implement both Random Forest and Naive Bayes algorithms, respectively. The Random Forest algorithm is based on the method described in 19. These packages are compared with the MSclassifier as the median-supplement approaches represent enhancements in these methods implemented in R. In addition, these provide an objective evaluation of the tool.

## Methods

### Implementation

The package implements median-supplement approaches to machine learning, robust machine learning techniques that have the advantage of supporting complete compliance efforts by not missing sensitive sub-datasets or allowing certain sub-datasets to escape the classification process when balancing overall datasets. They are applicable to datasets with unequal numbers of instances associated with each class (group).

***Median-supplement machine learning algorithms.*** They involve the following steps:

1. Find the median of each attribute among all the samples (instances).
2. Find the scalar multiplication of the median of each attribute and a corresponding column vector of an m by n matrix of uniformly distributed random numbers between 0 and 1. *m* is the difference between the numbers of groups of samples, and n is the number of attributes. These form a supplementary set.
3. The supplementary set is added to the expression profiles to form the new balanced, median-supplement data set.
4. Finally, classification models are inferred from the median-supplement data.

**Figure 1.**
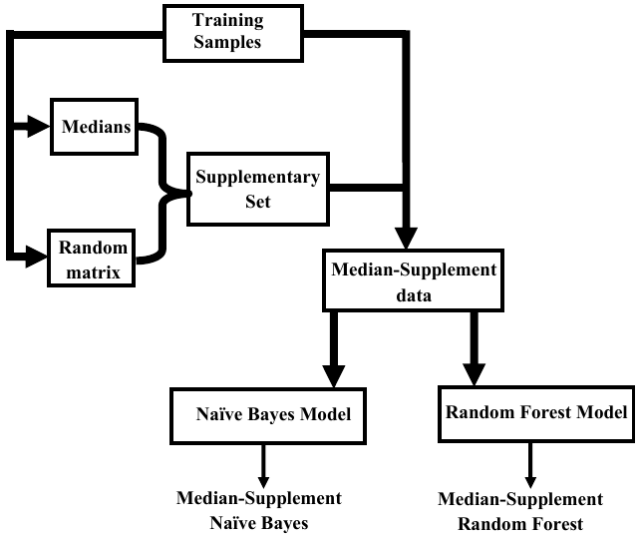
Overview of median-supplement models implementation in MSclassifier. A set of medians of attributes and a randomly generated matrix with uniformly distributed values are initially derived from the training sample. The result of a scalar multiplication of medians and corresponding column vectors of the random matrix is obtained and aggregated to the initial training sample to form a median-supplemented dataset. Finally, median-supplemented models are inferred from the median-supplemented data to predict new instances.

There are two kinds of median-supplement approaches, namely, median-supplement Random Forest and median-supplement Naive Bayes methods. Each approach is distinguished by the kind of model constructed from the median-supplement data. For a ’median-supplement Random Forest’, a Random Forest classifier is inferred from the median-supplement data to assign classes to instances. To obtain a ’median-supplement Naive Bayes classifier’, a Naive Bayes model is developed from the median-supplement data to classify instances. The overview of the underlying principles of median-supplement approaches as implemented in MSclassifier is shown in Figure 1.

***Naive Bayes model.*** This model applies the Bayesian framework to predict classes of new instances. Any classes having the highest posterior probability becomes the class of a new test instance. Let *G* be a set of attributes. Then, the probability that any instance belongs to any class/category, *C*_j_, is given by:

$$P(C_{j}|G)=\frac{P(G|C_{j})P(C_{j})}{P(G)},\;(1)$$

where *P(G|C _j_)* is the probability of *G* given class *C _j_, P(C _j_)* is the probability of *C _j_* and *P(G)* is the probability of *G* occurring. In this model, the attributes of each class are presumed to be independent distributions if the class is known. Thus, for each i-th attribute of *n* attributes, *g _i_*, the probability is given by:

$$P(G|C_{j})=P(g_{1}|C_{j})P(g_{2}|C_{j})\ldots P(g_{n}|C_{j})=\prod_{i=1}^{n}P(g_{i}|C_{j})\;(2)$$

***Random Forest model.*** Random Forest is advancement in multistage decision making. It is a collection of Decision Trees. This typically involves constructing a collection of trees from bootstrap samples each of which consists of a subset of variables of the training sets. This approach of inferring trees from bootstrap samples involves recursively repeating the following ^20^:

- Selecting *m* variables from the full set of attributes, *n,* at random *.*
- Selecting the best split among the variables.
- Split the nodes into two nodes.

Once all desired trees have been achieved in those steps (which repeats after reaching a putative node size), a classification is determined by a majority vote. Assume *C _b_(x)* is the class prediction of the b-th random forest tree. Then the classifier is given by:

$$C^{B}(x)=majorityvote{\left\{C_{b}(x)\right\}}_{1}^{B}.\;(3)$$

Typically, $m=\sqrt{n.}$ Using random forest spans from the fact that it improves predictive accuracies of tree-based methods ^19, 20^.

### Operation of MSclassifier

MSclassifier, implemented in R, can be installed and run on most operating systems. The sole requirement is the availability of a recent version of R ( https://cran.r-project.org/). The package is organized as a programme with the flexibility of selecting a median-supplement Random Forest or a median-supplement Naive Bayes method. The overview of the package follows the structure presented in Figure 1. The Documentation of the package has detailed instructions for installation and usage as well as other descriptions of the package.

MSclassifier does not require any special programming skills of the user. It accepts a tabular dataset in which the attributes and instances are in columns and rows respectively. In this way, the class of each instance is stored in the last column. At any time, two different datasets, training and test sets, may be supplied and the programme returns the predicted classes of instances of the test set. The training set comprises of characterized (labelled) samples whereas the test set is not characterized. In the absence of a test set, the user can specify only the training set to obtain a model for further analysis. Furthermore, the user specifies the desired median-supplement method. If no method is specified, a median-supplement Random Forest is automatically applied. Summary descriptions of arguments of MSclassifier function is described in Table 1. Samples of training and test sets are provided with the package. They are used in the illustration of the MSclassifier in the next section.

**Table 1.** Arguments (input parameters) of MSclassifier function.

Argument	Description
X	A data frame of values of attributes (e.g. gene expression levels) and classes (e.g. receptor status phenotypes in breast cancer). Samples are in rows while attributes are in columns. The last column of X should have the classes for all instances in X (e.g. receptor status phenotypes of samples). This form the training set.
Testset	This is the set of new instances to be classified. The Default is NULL. When set to NULL, the function returns only the model. To classify new instances, specify the data frame of the new instances as the test set. It should have the form (and attributes) of X.
Method	It specifies whether to determine a median-supplement Random Forest or median-supplement Naive Bayes. "MSRandomForest" infers median-supplement Random Forest. "MSNaiveBayes" applies the median-supplement Naive Bayes. The default is median-supplement Random Forest.

### Illustration (usage) of MSclassifier

In order to illustrate the use of the package, we use HER2 datasets included in the package. These datasets were obtained from an earlier study that explored the use of machine learning techniques to determine hormone and receptor status phenotypes in breast cancer ^17^. The training data consists of 86 HER2 receptor-negative and 14 HER2 receptor-positive samples while the independent test set consists of 51 HER2 receptor-negative samples and 11 HER2 receptor-positive samples. The illustration shows how to use the MSclassifier after installation.



Comprehensive description of function and application can be found in the help file after loading the installed package and getting the full description of the package:
> library(MSclassifier)  		# load package
> ?MSclassifier

To load package datasets:

> data(her2)
> data(testset)

To view a subset of the package’s her2 training set:
> her2[1:3,1:3]
      NPTXR_23467 DOCK3_1795 LOC400927_400927
1    266.0075         38.1356          12.7119
2    461.8575         34.9231           6.5481
3    199.3335         11.8146           6.9676

To view a subset of the package’s her2 test set:

> testset[1:3,1:3]
      NPTXR_23467  DOCK3_1795  LOC400927_400927
1    304.5058          21.0756           0.7267
2    453.4778          10.1620           7.5072
3    510.8080            3.5776           4.9292

To classify instances/determine her2 status of the test samples using median-supplement Random Forest, the following apply:

> Predictions <- MSclassifier(her2,testset = testset, method ="MSRandomForest")
> head(Predictions)
Sample1  Sample2  Sample3  Sample4  Sample5  Sample6
Negative Negative Positive Negative Negative Negative
Levels: Negative Positive

To analyse median-supplement Random Forest for error matrix, here is a sample:

 > Model <- MSclassifier(her2, testset = NULL, method = "MSRandomForest")
 > predictions <- predict(Model, newdata = testset)
 > head(predictions)
 1        2        3        4        5        6
 Negative Negative Positive Negative Negative Negative
 Levels: Negative Positive

 > table(predictions, testset$her2_status)
  predictions Negative Positive
  Negative       47          10
  Positive        4           1

 To classify instances/determine her2 status of the test samples using median-supplement Naive Bayes, here is a sample:

 > Predictions <- MSclassifier(her2,testset = testset, method = "MSNaiveBayes")
 > head(Predictions)
 Sample1  Sample2  Sample3  Sample4  Sample5  Sample6
 Negative Negative Positive Negative Negative Negative
 Levels: Negative Positive

To analyse median-supplement Naive Bayes for error (confusion) matrix, the following is an example:

> Model <- MSclassifier(her2, testset = NULL, method = "MSNaiveBayes")
> predictions <- predict(Model, newdata = testset[,-ncol(testset)])
> head(predictions)
[1] Negative Negative Positive Negative Negative Negative
Levels: Negative Positive

> table(predictions, testset$her2_status)
predictions Negative Positive
Negative       51          8
Positive        0          3


***Data sets.*** In order to illustrate the performance of the package, we use two real datasets. Particularly, the first data, obtained from previous study ^17^ describes gene expression measurements in breast cancer. In this illustration, median-supplement models are inferred with the MSclassifier package to assign classes to new instances of the test set. In the case of the HER2 data, the class of each instance is the expressed receptor status phenotype while attributes are the relevant gene expression profiles. The data consists of 86 HER2 receptor-negative and 14 HER2 receptor-positive samples. These are samples included in the MSclassifier package.

The second (larger) dataset was derived from a study that characterized amino acid sequences of human proteins localized in nine cellular compartments ^21^. Code written in LISP was used to determine values of physicochemical properties of proteins known to be primarily localized in the designated subcellular locations were used. Protein properties used are based on the amino acid composition (including hydrophobicity, normalized van der Waals volume, polarity, polarizability, and charge), transitions and distribution as detailed ^21^. For instance, "PERCENT-R" is a reference to the percentage of arginine residues in the primary sequence of amino acids of a protein; "HYDROPHOBICITY-PERCENT-GROUP1" is a reference to the percentage of polar amino acids in the primary sequence of amino acids (i.e. group 1 amino acids are polar, group 2 amino acids are neutral, and group 3 amino acids are hydrophobic); "POLARITY-GP1-GP3-TRANSITIONS" is a reference to the frequency of transitions between low polarity residues (L, I, F, W, C, M, V, and Y) and high polarity residues in a given protein’s primary sequence of amino acids (H, Q, R, K, N, E, D). The data comprised of 2635 instances and 126 attributes. Among the instances, 1589 were associated with (localized in) the plasma-membrane and 1046 were associated with the nucleus ^22^. In its usage to illustrate the package, instances of the dataset were classified as "nucleus" and "plasma-membrane".

***Performance measures of packages.*** The performance of each method is determined by its classification rate: proportion of correctly classifying instance given by the ratio of correctly classified test instances to the total number of test instances ^23^. In general, the classification rates agree with measures of accuracies of such classification methods. Higher classification rate of a method indicates that the package has higher chances of making accurate assignments of samples to their respective classes. Therefore, it is desirable to have higher classification rate. For instance, a higher classification rate for classifying receptor status phenotypes in breast cancer indicates the method has high sensitivity for deciphering the particular receptor status. This is because the sensitivity is also a proportion of correctly classified instances among characterized instances as exemplified in unsupervised learning systems ^24^. Furthermore, Mann-Whitney tests are performed to evaluate differences among classification rates of the methods. Both independent and cross-validation testing methods are used to evaluate the packages ^22^. While a 10-fold cross-validation is applied to the HER2 data, a 5-fold cross-validation is applied to the subcellular localization of proteins data ^22^.

## Results and discussion

### Performance on independent test sample

**Figure 2.**
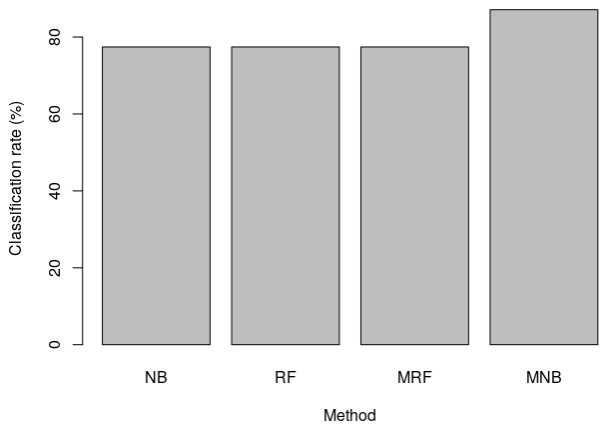
Comparison of MSclassifier and other packages in R. NB is Naive Bayes, RF is Random Forest, MRF is median-supplement Random Forest and MNB median-supplement Naive Bayes. While both MRF and MNB are implemented in MSclassifier, NB is implemented in e1071 and RF is implemented in randomForest packages.

In this experiment, HER2 training and test sets made available in the package were used. It was found that the median-supplement Naive Bayes (MNB) implemented in MSclassifier outperformed all the other methods considered in this case ( Figure 2). This was to be expected since the MSclassifier implements median-supplement methods, which have been shown to outperform the traditional machine learning methods ^17^. Higher performance of this package on this test example is the result of the enhanced median-supplement training set from which MSclassifier infers models. Thus the enhancement makes more instances available to train models.

### Performances from cross-validation testing

The classification rates of conventional methods, implemented in existing packages, ranged between 83% and 91%, methods implemented in the MSclassifier had values with minimum of 87% and maximum of 91%. Particularly, it was found that conventional random forest was significantly higher than the Naive Bayes (mean classification rate of 89.83% versus 85.43%, p = 1.48e-11). However, the median-supplement Naive Bayes implemented in MSclassifier achieved the highest classification rates among all the methods ^22^. More importantly, it had significantly higher classifications rates than the random forest method (mean is 90.30% versus 89.83%, p = 8.62e-3). These results are consistent with performance of median-supplement methods on HER2 classifications studied earlier ^17^.

With regards to the prediction of subcellular localization of proteins, although both MSclassifier and the other packages could attain equally high classification rates (94%) in this test, the minimum classification rate achieved by the median-supplement Naive Bayes was lower compared to the conventional Naive Bayes method (mean of 69% versus 86%, p = 4.55e-14). However, this observation was different in other studies ^17^. The difference is attributable to the differences in data and prediction tasks. Nevertheless, these performances are suboptimal when compared to the random forest-based methods which achieved mean classification rates of 93% ^22^. Specifically, the performances of both the random forest and the median-supplement random forest were statistically indistinguishable (mean of 93.42% versus 93.19%, p = 0.06). These results are indicative that tree-based random forest methods have better performances on larger datasets. However, the superiority of median-supplement methods over several other machine learning methods when applied to predict hormone and HER2 receptor phenotypes underpinned in the literature ^17^. These results demonstrate the potential of MSclassifier to better predict instances of binary classifications problems.

## Conclusion

We have presented the MSclassifier package to implement median-supplement approaches for machine learning to support medical decisions. The package was shown to decipher HER2 receptor status phenotypes in breast cancer and also predict subcellular localizations of proteins. MSclassifier compares favourably well with existing packages because it implements enhanced methods which offer effective approach to machine learning. Finally, MSclassifier can be installed and run on most operating systems. The sole requirement is the availability of a recent version of R. MSclassifier, steps for installation and other supplementary information are freely available at https://nweb.gimpa.edu.gh/schools/school-of-technology/software/MSclassifier/. Furthermore, the MSclassifier package and every other supporting data for this work have also been made publicly available at https://doi.org/10.5281/zenodo.3946675 ^22^.

## Software availability

Software available from: https://nweb.gimpa.edu.gh/schools/school-of-technology/software/MSclassifier/

Source code available from: https://github.com/esadabor/MSclassifier.git

Archived source code as at time of publication: https://doi.org/10.5281/zenodo.3946675 ^22^

License: GPL-3

## Data availability

### Underlying data

Datasets used in Use Case:

- Gene expression measurements in breast cancer, obtained from previous study ^17^. Dataset available here: http://doi.org/10.5281/zenodo.3964514 ^25^ (permission to reuse this dataset and to republish on Zenodo has been granted by Oxford University Press).
- Characterized amino acid sequences of human proteins localized in nine cellular compartments, obtained from previous study ^21^. Dataset available here: http://doi.org/10.5281/zenodo.3964503 ^26^. Protein subcellular localisation dataset available here: https://doi.org/10.5281/zenodo.3946675 ^22^.

### Extended data

Zenodo: Supporting information and data for MSclassifier: Median-Supplement model-based Classification tool for automated knowledge discovery, https://doi.org/10.5281/zenodo.3946675 ^22^.

This project contains the following extended data:

- Cross-Validation Testing information
- Table S2: Performance of methods on HER2 dataset
- Table S3: Performance of methods on plasma-membrane and nucleus classification dataset
