# Supplementary material for: MSclassifier: median-supplement model-based classification tool for automated knowledge discovery
Source: F1000Res. 2020 Sep 10;9:1114. [Version 1] doi: 10.12688/f1000research.25501.1 (PMC7788522; doi:10.12688/f1000research.25501.1)
Supplement: Peer review history

# Peer review: Reviewer response for version 1

**DOI:** 10.5256/f1000research.28142.r76091

**Published:** 2021-01-06

**Authors:** Alper Uzun

**Affiliations:** 1 Department of Pediatrics, Warren Alpert Medical School of Brown University, Providence, RI, USA

The authors developed a median-supplement based tool, MSclassifier to automate knowledge discovery from available data sets. They demonstrated that it can be applied to binary classification problems. In this regard they used two datasets to demonstrate their application. The authors implemented the application in R which is freely accessible.

 I recommend the manuscript for indexing with minor revision:

 - Area Under the Receiver Operator Characteristic (AUROC) is a very useful metric to evaluate models. There are packages available to use AUROC. The manuscript will be benefited by adding this metric into the results.

Are the conclusions about the tool and its performance adequately supported by the findings presented in the article?

Yes

Is the rationale for developing the new software tool clearly explained?

Yes

Is the description of the software tool technically sound?

Yes

Are sufficient details of the code, methods and analysis (if applicable) provided to allow replication of the software development and its use by others?

Yes

Is sufficient information provided to allow interpretation of the expected output datasets and any results generated using the tool?

Yes

Reviewer Expertise:

I focus on using genomics and bioinformatics to understand the genetic architecture of complex diseases. Our research group is managing large scale data (whole-genome genotyping data, RNA-Seq, targeted sequencing) and developing bioinformatics methods to identify causal variants. We use machine-learning approach to extract disease associated genes from published biomedical literature. We develop bioinformatics tools for both analyzing and visualizing protein protein interaction networks and in addition we develop tools to visualize variants.

I confirm that I have read this submission and believe that I have an appropriate level of expertise to confirm that it is of an acceptable scientific standard.

# Peer review: Reviewer response for version 1

**DOI:** 10.5256/f1000research.28142.r74679

**Published:** 2021-01-06

**Authors:** Gregory Hart

**Affiliations:** 1 Department of Therapeutic Radiology, Yale University, New Haven, CT, USA

I would suggest that (and assume it would be easy) you allow for your package to run regular Random Forest and Naive Bayes in addition to the MS versions. While that functionality is already available in other packages it would be nice to have it all in one place.

 In addition to the accuracy comparison it would be nice to get a feel for the computational cost of adding MS as well as how the algorithm scales.

 Lastly, any reason to only do Random Forest and Naive Bayes? Would there be interest in including other classifiers in the future?

Are the conclusions about the tool and its performance adequately supported by the findings presented in the article?

Partly

Is the rationale for developing the new software tool clearly explained?

Yes

Is the description of the software tool technically sound?

Yes

Are sufficient details of the code, methods and analysis (if applicable) provided to allow replication of the software development and its use by others?

Yes

Is sufficient information provided to allow interpretation of the expected output datasets and any results generated using the tool?

Partly

Reviewer Expertise:

My postdoc was focused on machine learning and applying it to cancer diagnosis. The biology/medicine is often over my head by the software/methods are not.

I confirm that I have read this submission and believe that I have an appropriate level of expertise to confirm that it is of an acceptable scientific standard.
